# Mapping knowledge structure and themes trends in unilateral biportal endoscopic spine surgery: A bibliometric analysis

**DOI:** 10.3389/fsurg.2022.976708

**Published:** 2022-09-07

**Authors:** Ming-Tao Zhu, Kunrong Li, Bao-Shan Hu, Chien-Min Chen, Guang-Xun Lin

**Affiliations:** ^1^ Department of Neurosurgery, The First Affiliated Hospital of Xiamen University, School of Medicine, Xiamen University, Xiamen, China; ^2^ Department of Orthopedics, The First Affiliated Hospital of Xiamen University, School of Medicine, Xiamen University, Xiamen, China; ^3^ Division of Neurosurgery, Department of Surgery, Changhua Christian Hospital, Changhua, Taiwan; ^4^ Department of Leisure Industry Management, National Chin-Yi University of Technology, Taichung, Taiwan; ^5^ School of Medicine, Kaohsiung Medical University, Kaohsiung, Taiwan; ^6^ The Third Clinical Medical College, Fujian Medical University, Fuzhou, China

**Keywords:** unilateral biportal endoscopic, biportal endoscopic spine surgery, bibliometric analysis, visualization, research trends

## Abstract

**Background:**

The numerous benefits of unilateral biportal endoscopic (UBE) spine surgery have attracted the attention of many researchers, and a considerable number of relevant clinical studies have been published. However, global research trends in the field of UBE have received little attention. The purpose of this study was to apply bibliometric method to analyze the UBE-related publications to obtain an overview of the research trends in the field of UBE, as well as research hotspots and trends.

**Methods:**

Web of Science database was searched for articles published until January 31, 2022. CiteSpace was used to analyze the data, which provided graphical knowledge maps. The following factors were applied to all literature: number of publications, distribution, h-index, institutions, journals, authors, and keywords.

**Results:**

Seventy-three articles were identified. Since 2019, there has been a significant increase in the number of UBE-related publications. The country with the largest number of articles was South Korea (72.6%), followed by China (9.6%), Japan (4.1%), and Egypt (4.1%). South Korea had the highest h-index (16), followed by China (2), Japan (1), and Egypt (1). Leon Wiltse Memorial Hospital was the organization that produced the most papers (12 publications). Heo DH was the most productive author (16 papers) and was the most cited author (35 times). *World Neurosurgery* published the most papers on UBE (23.3%). The main research hotspots were spinal diseases, decompression, complications, learning curve, and interbody fusion. In addition, the recent concerns were “learning curve,” “interbody fusion,” “management,” and “dural tear.”

**Conclusions:**

The quantity of publications on UBE research will increase, and South Korea being the major contributor and most prominent country in this field. The findings of our study will provide researchers with practical information on the field of UBE, and identification of mainstream research directions and recent hotspots.

## Introduction

With the accelerating trend of aging in society and changes in people's lifestyle and work style, the incidence of lumbar spinal diseases is gradually increasing. For patients requiring surgical treatment, traditional open surgery is highly traumatic and has many complications, and microendoscopic techniques have certain complications that do not fully meet the requirements of patients (1). The unilateral biportal endoscopic (UBE) technique is an emerging clinical treatment tool with the advantages of a wide surgical field of view and large operating space, which can be implemented *via* the interlaminar or transforaminal approach and successfully applied in treating various spinal surgical diseases (2–4). As a minimally invasive surgery, it combines the advantages of open surgery and traditional minimally invasive surgery, preserving the paravertebral muscles while operating under high-definition vision, reducing damage to the paravertebral bones, joints, and ligaments, with the advantages of less postoperative pain and early return to normal activities, and is therefore widely used in treating various spinal disorders (5–7).

As a new technique, UBE has attracted the attention of many researchers and a large number of clinical studies have been published recently. A bibliometric analysis can provide clinical researchers with practical information, including the influential countries/regions, journals, institutions, and authors in the field (8). In addition, bibliometrics helps comprehend a topic's underlying knowledge, current research hotspots, and research trends (9).

Therefore, this bibliometric study aims to analyze the published UBE-related literature to obtain an overview of the current status and trends of UBE research and to provide recommendations and suggestions for the development of related research in the future.

## Materials and methods

### Search strategy

Because this was a retroactive assessment of public data, no institutional committee permission was necessary. Publications were gathered from the Web of Science (WoS) Core Collection (Thomson Reuters, New York, NY, USA), which is the world's biggest academic database and has been frequently used in bibliometric research.

The publications were evaluated until January 31, 2022. The following terms were searched: “biportal endoscopic spine surgery,” “unilateral biportal endoscopic surgery,” “UBE,” “BESS,” and “two portal endoscopic spine surgery.” Only original articles, reviews, and case reports were accepted; letters, editorial materials, and corrections, as well as unpublished and non-English studies, were excluded from this study. In addition, documents on unrelated topics were excluded. Two researchers independently reviewed and chose the publications. Any disagreements were resolved through third-party discussions until consensus was reached.

To conclude the bibliometric investigation, we deployed CiteSpace to construct data tables and visual knowledge graphs for interpretation. CiteSpace is essentially built on the concept of co-citation analysis and pathfinder network scaling to evaluate the literature in a certain field so that users may discover significant advances and knowledge turning points in the discipline's history (10).

Our quantitative studies were based on the number of publications each year, nations/regions, the h-index (a legitimate and trustworthy measure for academic assessment), institutions, journals, authors, citations, and keywords. In the present study, CiteSpace was used to conduct a cooperative analysis of regions, institutions, and authors; to perform the impact of scientific journals; to analyze the top 10 most cited documents; and to identify the top 10 keywords with the strongest citation bursts. The node connection represented a relationship of cooperation, co-occurrence, or co-citation in the network maps. The links in the visualization knowledge maps between nodes reflected the cooperative ties. The thickness of the linkages and the distance between the nodes showed the extent to which prominent nations/regions, institutions, and writers collaborated.

### Data examination

All data were gathered and entered into Microsoft Excel 2021 (Microsoft). CiteSpace was used to quantify data, display cooperation networks in various layouts, and create a term timeline.

## Results

### Annual trend and current situation

**Figure 1:**
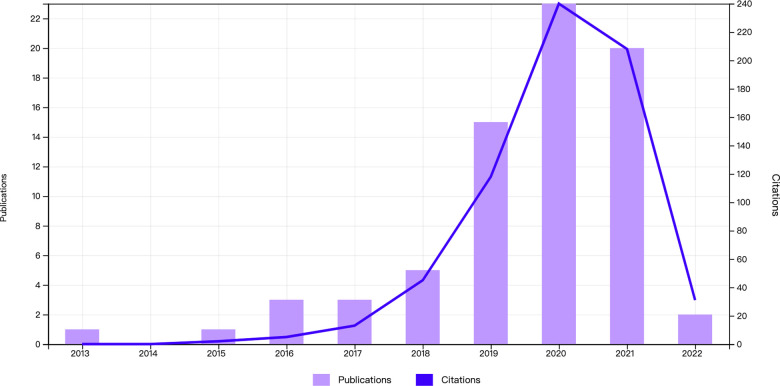
The annual trends of publications.

Initially, the WoS database contained 89 articles on the subject of UBE. Seventy-three publications were chosen after manual screening. The number of publications in the field of UBE rapidly rose in the past 3 years (Figure 1). In 2020, 23 articles were published; this number was the most in a single year in the previous decade. The number of publications about UBE is steadily rising, indicating that more attempts and explorations in UBE are being made.

### Analysis of countries/regions

**Figure 2:**
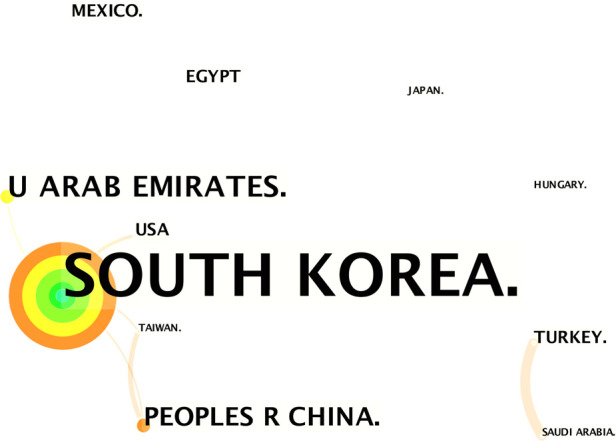
Co-operation network of the productive countries/regions.

In the field of UBE, 10 countries have conducted studies throughout the study period (Table 1). South Korea produced the most publications (53 of 73, 72.6%), followed by China (7 of 73, 9.6%), Japan (3 of 73, 4.1%), and Egypt (3 of 73, 4.1%). South Korea has the highest h-index at 16, followed by China (2), Japan (1), and Egypt (1). The map of the country's network had 40 nodes and 40 links (Figure 2).

**Table 1:** The most productive countries/regions contributed to research publications in the field of unilateral biportal endoscopic spine surgery.

Rank	Country	Number	Percentage	h-Index
1	South Korea	53	72.6	16
2	China	7	9.6	2
3	Japan	3	4.1	1
4	Egypt	3	4.1	1

### Analysis of institutions

**Figure 3:**
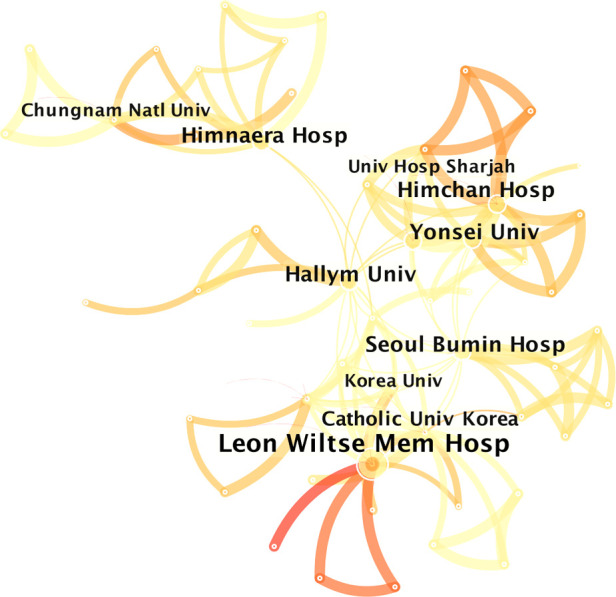
Co-operation network of the productive institutions.

Table 2 shows the most productive institutions in the field of UBE. Of the 73 publications, Leon Wiltse Memorial Hospital published 12 articles (16.4%), followed by Hallym University, which published 10 articles (13.7%); Himchan Hospital, which published seven articles (9.6%); and Himnaera Hospital, Seoul Bumin Hospital, and Yonsei University, which published seven articles (9.6%). The institution network map has 81 nodes and 139 connections (Figure 3).

**Table 2:** The most productive institutions in the field of unilateral biportal endoscopic spine surgery.

Rank	Institution	Number	Percentage
1	Leon Wiltse Memorial Hospital	12	16.4
2	Hallym University	10	13.7
3	Himchan Hospital	7	9.6
4	Himnaera Hospital	7	9.6
5	Seoul Bumin Hospital	7	9.6
6	Yonsei University	7	9.6

### Analysis of journals

UBE was featured in 25 scientific journals during the research period. *World Neurosurgery* published the most articles regarding UBE (17 articles, 23.3%) (Table 3), followed by *Acta Neurochirurgica* (9 articles, 12.3%), *Journal of Orthopaedic Surgery and Research* (5 articles, 6.8%), *Neurospine* (5 articles, 6.8%), and *Spine Journal* (5 articles, 6.8%). Articles published in these essential journals received more attention and therefore were referenced more frequently.

**Table 3:** Top 5 productive journals in the field of unilateral biportal endoscopic spine surgery.

Rank	Journal	Number	Percentage
1	World Neurosurgery	17	23.3
2	Acta Neurochirurgica	9	12.3
3	Journal of Orthopaedic Surgery and Research	5	6.8
4	Neurospine	5	6.8
5	Spine Journal	5	6.8

### Analysis of authors

**Figure 4:**
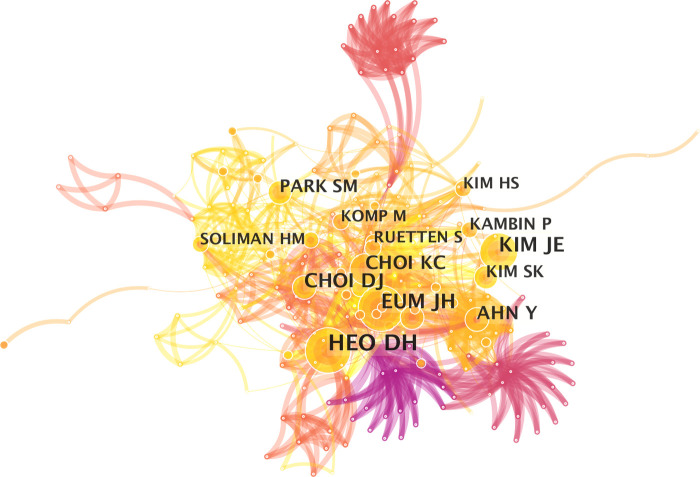
Co-operation network of the cited authors.

Heo DH was the most productive author in the field of UBE (Table 4), publishing 16 articles (21.9%); Choi DJ published 11 articles (15.1%), Park CK published 10 articles (13.7%), Chung HJ published 9 articles (12.3%), and Park HJ wrote 9 articles (12.3%). The cited author network's map has 263 nodes and 1,080 linkages (Figure 4). The most frequently cited author (35 times) was Heo DH, followed by Eum JH (26 times), Kim JE (25 times), Choi DJ (22 times), and Choi KC (18 times).

**Table 4:** Top 5 productive authors in the field of unilateral biportal endoscopic spine surgery.

Rank	Author	Number	Percentage	Affiliation
1	Heo DH	16	21.9	Leon Wiltse Memorial Hospital, South Korea; Seoul Bumin Hospital, South Korea
2	Choi DJ	11	15.1	Himnaera Hospital, South Korea
3	Park CK	10	13.7	Leon Wiltse Memorial Hospital, South Korea
4	Chung HJ	9	12.3	Seoul Bumin Hospital, South Korea
5	Park HJ	9	12.3	Hallym University, South Korea

### Analysis of references and citations

**Figure 5:**
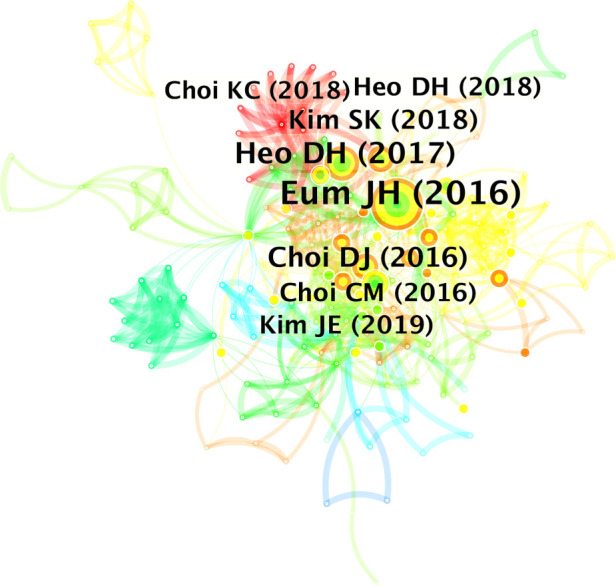
Co-operation network of the cited references.

The top ten most cited articles are presented in Table 5. An article's highest and lowest numbers of citations were 74 and 22, respectively. Nine of the top 10 most cited articles were from South Korea, whereas the remaining article was from Egypt. Heo DH has four articles on this list. Three articles were published in *World Neurosurgery* or *Neurosurgical Focus*, respectively. The network map of the references mentioned has 181 nodes and 744 linkages (Figure 5). The top 5 most frequently referenced article was Eum JH et al. (2016) (26 times), followed by Heo DH et al. (2017) (18 times), Choi DJ et al. (2016) (15 times), Kim SK et al. (2018) (13 times), and Choi CM et al. (2016) (12 times).

**Table 5:** Top 10 cited articles in the field of unilateral biportal endoscopic spine surgery.

Rank	Title	Author	Journal	Year	Citation
1	Percutaneous biportal endoscopic decompression for lumbar spinal stenosis: a technical note and preliminary clinical results	Eum JH et al.	Journal of Neurosurgery-Spine	2016	74
2	Fully endoscopic lumbar interbody fusion using a percutaneous unilateral biportal endoscopic technique: technical note and preliminary clinical results	Heo DH et al.	Neurosurgical Focus	2017	68
3	How I do it? Biportal endoscopic spinal surgery (BESS) for treatment of lumbar spinal stenosis	Choi CM et al.	Acta Neurochirurgica	2016	36
4	Irrigation endoscopic decompressive laminotomy	Soliman HM et al.	Spine Journal	2015	36
5	Comparison of surgical invasiveness between microdiscectomy and 3 different endoscopic discectomy techniques for lumbar disc herniation	Choi KC et al.	World Neurosurgery	2018	31
6	Can percutaneous biportal endoscopic surgery achieve enough canal decompression for degenerative lumbar stenosis? Prospective case-control study	Heo DH et al.	World Neurosurgery	2018	30
7	Learning curve for lumbar decompressive laminectomy in biportal endoscopic spinal surgery using the cumulative summation test for learning curve	Park SM et al.	World Neurosurgery	2019	27
8	Comparative analysis of three types of minimally invasive decompressive surgery for lumbar central stenosis: biportal endoscopy, uniportal endoscopy, and microsurgery	Heo DH et al.	Neurosurgical Focus	2019	26
9	Clinical results of percutaneous biportal endoscopic lumbar interbody fusion with application of enhanced recovery after surgery	Heo DH et al.	Neurosurgical Focus	2019	23
10	Biportal endoscopic versus microscopic lumbar decompressive laminectomy in patients with spinal stenosis: a randomized controlled trial	Park SM et al.	Spine Journal	2020	22

### Analysis of keywords and research hotspots

**Figure 6:**
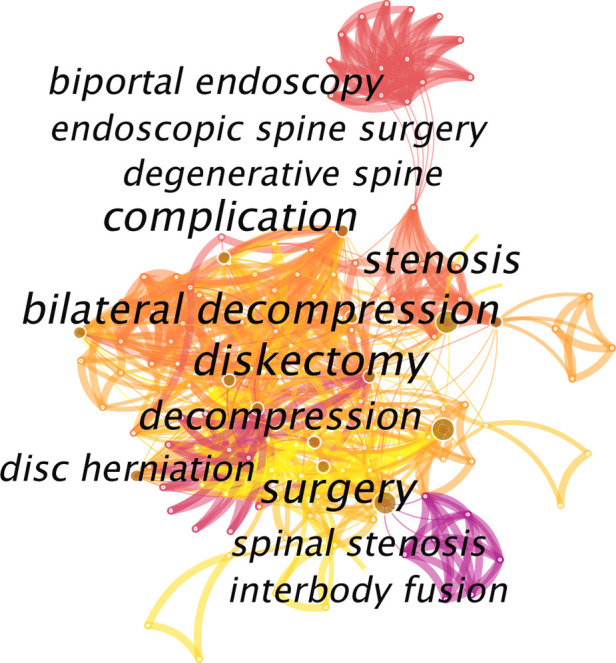
Co-operation network of the keywords.

Keyword lists can effectively discover research hotspots and provide research assistance. Bigger nodes in the keyword co-occurrence map had larger keyword weights. Shorter distances between nodes suggested stronger connections between those nodes. Thicker lines indicated a higher frequency of two words being mentioned together. As shown in Figure 6, the main research hotspots were as follows: spinal diseases, decompression, complications, learning curve, and interbody fusion.

**Figure 7:**
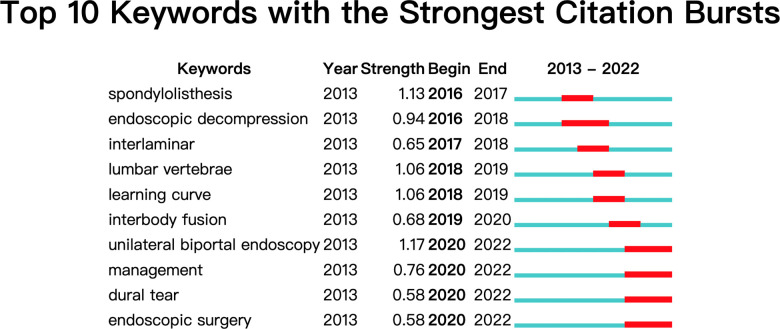
Top 10 keywords with the strongest citation bursts.

“Keyword bursts” were an indication of research frontier themes throughout a certain period. Figure 7 shown the top 10 keywords with the strongest citation bursts in the UBE field. The red bar corresponded to the time of keyword appearance and duration of presence. The recent concerns were “learning curve,” “interbody fusion,” “management,” and “dural tear.”

## Discussion

UBE is a percutaneous endoscopic technique that uses two channels, one for endoscopy and one for instrumentation, which is the major difference from the traditional single-portal endoscopic technique (11, 12). The UBE procedure is mainly used for endoscopic treatment of spinal stenosis, cervical spondylosis, thoracic spine lesions, and degenerative lesions of the lumbar spine (13–15). Since two channels are used, the operating instruments are not limited in size; thus, the UBE technique is an efficient technique among various minimally invasive spine techniques, and the treatment results are as thorough as those of open surgery, with certainty of efficacy, less trauma, and faster recovery (16, 17). Additionally, traditional single-portal endoscopic techniques can address a small percentage of spinal stenosis cases, a large percentage of which are not operable, and the UBE technique with unilateral dual access can better address cases of spinal stenosis. The UBE technique is complementary to the single-portal endoscopic technique and can be used for partial vertebral instability and minor slippage, as well as for endoscopic spinal fusion, which has a broader range of indications (12).

The merits of the UBE technique have brought more attention to spine surgeons, as evidenced by the increase in clinical studies in recent years. To confirm the comprehensiveness of the publications, we conducted a bibliometric study of publications in the WOS database. Our results will provide researchers with practical information on the field of UBE, and identification of mainstream research directions and recent hotspots.

This study found a consistent increase in the quantity of UBE-related publications recently, particularly after 2019. This pattern demonstrates that UBE research is advancing quickly and has piqued the interest of the worldwide medical community. South Korea is the most productive country in the UBE field and has published the most articles and is home to almost all influential authors. Moreover, nine of the top 10 most cited articles were from South Korea. Early scholars, represented by Dr. Kambin, developed the percutaneous spinal endoscopy technique, followed by their predecessors, such as De Antoni DJ and Osman SG, who laid the theoretical and practical foundation for the “unilateral biportal spinal endoscopy” technique. Although the studies by De Antoni DJ and Osman SG have inspired some operators to trace their concepts and findings, this group of operators has embarked on a path to continue exploring dual-channel spinal endoscopy techniques. In the next decade, the unilateral biportal endoscopic technique will enter a period of rapid development driven by Korean spine surgeons, and many improvements were made, as follows: (1) changing the patient's position from lateral to prone; (2) starting to use radiofrequency, which improved the efficiency of handling soft tissue; (3) further expanding the indications for the procedure, adding spinal disk herniation, spinal stenosis, spondylolisthesis, and fusion (cervical-thoracic-lumbar spine can be applied); and (4) formalizing the procedure as UBE. The contribution of Korean doctors to the inheritance, pioneering, and development of the UBE technique has made them well known internationally. This has led several spine surgeons to go to South Korea for further training and study.

According to the network map, countries/regions, institutions, and authors were all somewhat connected; however, the map shows a weak relationship, indicating a lack of cooperation between countries/regions and institutions. International academic cooperation between countries/regions and institutions must be strengthened. This technique may benefit all countries/regions and institutions.

*World Neurosurgery*, *Acta Neurochirurgica*, *Journal of Orthopaedic Surgery and Research*, *Neurospine*, and *Spine Journal* were the top five journals that have published the most UBE-related articles, suggesting that these journals are more friendly to the publication of UBE-related articles. The 10 most cited articles were from the aforementioned journals, with *World Neurosurgery* and *Neurosurgical Focus* being the top two journals. These journals represented the core journals in the field of UBE and should be followed to track relevant research trends.

Keywords not only represent the research focus and hotspots in a field, but also allow the discovery of research trends through keywords. According to top 10 keywords with the strongest citation bursts, the focus of UBE research includes the use of UBE in treating various lumbar spine diseases, prevention and treatment of complications, interbody fusion, and learning curve. UBE has recently evolved as a prominent lumbar surgical method; however, it must be used with objectivity and prudence to maximize its advantages and avoid its risks.

The UBE technique has more obvious technical advantages than the one-portal endoscopic surgical method. First, the UBE technique provides a larger and more open field of view under the mirror because the UBE procedure has a dual channel: one side of the main mirror is under 0°, the mirror field of view is 360° visible. Second, the grasping forceps and biting forceps used are thicker and have larger openings, which can remove the protruding nucleus pulposus faster and easier (18). The third reason is that the UBE technique can be visualized and the learning curve is relatively simple, especially if the surgeon can operate a one-portal endoscopic surgery or has experience in microscopic surgery. In addition, if the fusion is done under the endoscopy, the UBE technology can be visualized throughout the operation, and interbody cage placed directly under visualization, which greatly reduces the issue of intraoperative localization and radiation. In contrast, the single-portal technique for placing interbody cage is not visualizable and has a higher number of intraoperative localizations (19–21). A recent meta-analysis (6) has reported no significant differences in visual analog scale scores for the legs, Oswestry Disability Index scores, complications, or fusion rates between UBE interbody fusion and conventional lumbar interbody fusion surgery. Notably, the UBE interbody fusion surgical technique had considerably lower postoperative visual analog scale values for back pain than the traditional lumbar interbody fusion surgery. Furthermore, UBE interbody fusion took a longer operating time than traditional lumbar interbody fusion surgery but resulted in much less blood loss (6).

The most common complications of UBE were dural tears and hematomas, which were consistent with the findings of a previous systematic study (22, 23). A rupture in the dura is a serious issue. Endoscopic surgery may be converted to microsurgery in situations of large-scale dura ruptures. Small intraoperative durotomies can be sutured using sealant materials (TachoComb or TachoSil), and the patient should be restrained (3). The most important step in lowering the occurrence of this technical issue is to keep the operation field free by preventing epidural bleeding. A high magnification of the surgical field combined with continuous saline irrigation can be used to decrease epidural hematoma. When we started removing the flavum or conducting laminectomy, we needed to ensure that there was enough water flow and bleeding control, especially on the contralateral side (24). If all other measures fail to halt the bleeding, lowering the diastolic blood pressure to approximately 100 mmHg may be effective in certain cases (24). When raising the height of the saline bag or compressing it to raise the saline pressure, using a specialized pressure pump is advised. Moreover, high-pressure irrigation is not recommended because it may increase intracranial pressure and may delay surgical recovery. Water pressure should be maintained between 4.41 cm H_2_O (2.41 mmHg) and 31.00 cm H_2_O (22.83 mmHg) during UBE to avoid iatrogenic damage (25). Furthermore, preoperative anticoagulant use, female sex, elderly age, intraoperative water infusion pump use, and surgery involving higher bone manipulation were risk factors for epidural hematoma following UBE (26, 27).

## Limitations

To guarantee fairness and thoroughness and provide powerful data, we did a comprehensive literature search in the WoS datable. Despite its impressive characteristics, this study has a few flaws. First, this bibliometric analysis only included published articles from the WoS Core Collection database, which inevitably led to some useful literature not included in this study. Second, different search time points may have caused differences in the search results, especially in the number of citations. Third, for some recently published articles, the short time of publication leads to low citation counts, which may affect the total number of citations and h-index of the literature.

## Conclusions

According to our results, there was a dramatic increase in the number of UBE-related publications since 2019. Most of the UBE-related research institutions and researchers are from South Korea. Heo DH is the most contributing author, and Leon Wiltse Memorial Hospital has the largest contribution in this field. *World Neurosurgery* and *Neurosurgery Focus* represented the core journals in the field of UBE and should be followed to track relevant research trends. The main research hotspots in the field of UBE were the use of UBE in treating various lumbar spine diseases, prevention and treatment of complications, interbody fusion, and learning curve.

## Data Availability

The original contributions presented in the study are included in the article/Supplementary Material, further inquiries can be directed to the corresponding author/s.
